# Indications of strong adaptive population genetic structure in albacore tuna (*Thunnus alalunga*) in the southwest and central Pacific Ocean

**DOI:** 10.1002/ece3.5554

**Published:** 2019-08-27

**Authors:** Giulia Anderson, John Hampton, Neville Smith, Ciro Rico

**Affiliations:** ^1^ School of Marine Studies Molecular Analytics Laboratory (MOANA‐LAB) Faculty of Science Technology and Environment The University of the South Pacific Suva Fiji; ^2^ Oceanic Fisheries Programme (OFP) Pacific Community Nouméa New Caledonia; ^3^ Instituto de Ciencias Marinas de Andalucía (ICMAN) Consejo Superior de Investigaciones Científicas Cádiz Spain

**Keywords:** adaptive genetic structure, life stage, single nucleotide polymorphism, *Thunnus alalunga*

## Abstract

Albacore tuna (*Thunnus alalunga*) has a distinctly complex life history in which juveniles and adults separate geographically but at times inhabit the same spaces sequentially. The species also migrates long distances and presumably experiences varied regimes of physical stress over a lifetime. There are, therefore, many opportunities for population structure to arise based on stochastic differences or environmental factors that promote local adaptation. However, with the extent of mobility consistently demonstrated by tagged individuals, there is also a strong argument for panmixia within an ocean basin. It is important to confirm such assumptions from a population genetics standpoint for this species in particular because albacore is one of the principal market tuna species that sustains massive global fisheries and yet is also a slow‐growing temperate tuna. Consequently, we used 1,837 neutral SNP loci and 89 loci under potential selection to analyze population genetic structure among five sample groups collected from the western and central South Pacific. We found no evidence to challenge panmixia at neutral loci, but strong indications of structuring at adaptive loci. One population sample, from French Polynesia in 2004, was particularly differentiated. Unfortunately, the current study cannot infer whether the divergence is geographic or temporal, or possibly caused by sample distribution. We encourage future studies to include potentially adaptive loci and to continue fine‐scale observations within an ocean basin, and not to assume genome‐wide panmixia.

## Graphical abstract

High‐throughput NGS sequencing identified 1,837 neutral loci and 89 potentially adaptive loci among albacore tuna from five sample groups collected in the western and central South Pacific. We found panmixia at neutral loci, but unexpectedly high population differentiation at adaptive loci between western and central Pacific locations. Although we cannot confirm whether this indicates spatial, temporal, or life‐stage population genetic structure, the findings suggest albacore population structure deserves much more study than is currently allocated.

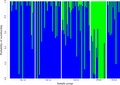

## 1 INTRODUCTION

**Figure 1:**
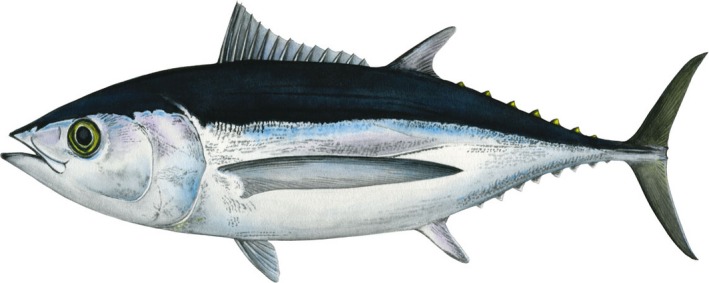
Albacore tuna, *Thunnus alalunga*. Illustration: Les Hata, © Pacific Community

Albacore tuna (*Thunnus alalunga*) (Figure 1) fills an unusual niche among economically important tuna species. Skipjack, yellowfin, and bigeye tuna all outpace albacore by fishery volume, and bluefin species all fetch much higher prices per weight unit. However, at more than 232,000 mt in 2017 (Tuna Fishery Yearbook 2017, 2018) and only $14,000 USD per tonne for fresh, sashimi‐grade meat (Macfadyen & Defaux, 2016), albacore fills a significant niche of consumer demand for quality protein at moderate prices. Unfortunately, unlike tropical tunas that can sustain large volume extraction industries due to their high fecundity and rapid growth to maturity (Goujon & Majkowski, 2000), albacore takes four or more years to mature (Duncan, Brophy, & Arrizabalaga, 2018; Williams, Farley, Hoyle, Davies, & Nicol, 2012). This slow growth pattern makes albacore, like bluefin and other temperate tuna species, much more susceptible to overfishing than tropical counterparts (Murua, Rodriguez‐Marin, Neilson, Farley, & Juan‐Jordá, 2017).

There is no evidence of current overfishing of albacore tuna in any ocean basin (ISSF, 2017), but there is also no comprehensive study that assesses all phases of the species' complex life history. Fish in the Indian and South Pacific Ocean spawn in the tropics during the spring and summer months and make their first migration to high latitudes largely undetected, appearing around 40°S roughly a year later (Farley, Williams, Hoyle, Davies, & Nicol, 2013). Juveniles persist at high latitudes until they reach sexual maturity and in the meantime follow smaller summer migrations to feeding grounds at latitudes of 15–25°S (Chen, Lee, & Tzeng, 2005). Once they become sexually mature, fish move closer to the equator, now wintering in the same latitudes that become juvenile feeding grounds in the summer, and migrating in the spring into tropical waters <15°S in order to spawn (Chen et al., 2005). Apart from the failure to mark yearly recruits during their first migration to 30°S, most elements of the division between life phases have been well recorded through catch per unit effort and tagging data.

However, other aspects of the albacore life cycle are not completely understood (Hoyle & Davies, 2009; Nikolic et al., 2013) or described for all stocks. There is a much less well‐documented longitudinal element to albacore migrations, with young fish wintering offshore and migrating east to feed in both the North Pacific (Childers, Snyder, & Kohin, 2011) and North Atlantic (Arrizabalaga, López‐Rodas, Ortiz de Zárate, Costas, & Gonzaléz‐Garcés, 2002), and mature fish going from the same longitudes west to spawn (Arrizabalaga et al., 2002; Chen et al., 2005). Another juvenile feeding ground off New Zealand in the southwest Pacific is well recognized, but the corresponding spawning ground for the hemisphere is not confidently identified. Inferences by various studies place the spawning ground anywhere from the west Pacific to as far east as French Polynesia (Farley et al., 2014; Hoyle, Hampton, & Davies, 2012). The actual route taken by a single fish can also vary between years, both in how far offshore or toward the continental shelf they travel and the latitude to which they return (Childers et al., 2011; Duncan et al., 2018). Likewise, the longitudinal boundaries of these wintering grounds are not mutually agreed upon; tagging studies in the northeastern Pacific describe endpoints of migrations extending to between 130 and 180°W (Childers et al., 2011).

The story is even less complete from a population genetics perspective. Many studies have been successful in recognizing the differentiation of the Pacific, Indian, Atlantic, and Mediterranean populations, but putatively neutral loci rarely demonstrate differentiation within an ocean basin (Chow & Ushiama, 1995; Davies, Gosling, Was, Brophy, & Tysklind, 2011; Laconcha et al., 2015). This contradicts tagging studies (Arrizabalaga et al., 2002) and current management boundaries (Collette, Acero, Amorim, & Boustany, 2011), which suggest that there is no significant interaction between populations from different hemispheres within the same body of water. Loci flagged as possibly being under divergent selection (loci under potential selection, LUPS) are consistently more successful than neutral loci at demonstrating structure (e.g., Grewe et al., 2015) but are rarely employed and, like neutral loci, may be limited by study design. Specifically, studies' sample collection protocols rarely acknowledge the geographic separation of age groups or the different placement of age groups between seasons. Therefore, many forms of cryptic structure could exist that are not currently considered in the literature.

The importance of LUPS to determining management policies is debatable. Loci are only confirmed to be adaptive if they can be mapped to known genes in a reference genome and demonstrated to impact the downstream fitness of an individual (Pardo‐Diaz, Salazar, & Jiggins, 2015). Loci under strong selective constraints are extremely informative for management by identifying populations that are locally adapted under divergent environmental factors, which should not be translocated or infused with individuals from different demes (Frankham et al., 2011). Studies of species without an annotated reference genome traditionally use potentially selective loci in place of adaptive loci. LUPS are identified based on deviation from normal patterns of allele frequencies across multiple populations (Refoyo‐Martínez et al., 2019). More recently, Random Forest machine learning algorithm has been used to select loci that potentially interact and produce significant population discrimination despite unremarkable allele frequencies per individual locus (Brieuc, Waters, Drinan, & Naish, 2018; Jacobs et al., 2018). In both cases, panels of loci were specifically selected to produce differentiation patterns during population structure analyses that are otherwise overwhelmed by the signal of characteristically neutral loci. However, the adaptive status of loci selected in either fashion is still unconfirmed, and the biological relevance of downstream trends is likewise putative. The confidence of LUPS identification is also impacted by the number of sample groups observed, the true number of demes under observation, extent of neutral differentiation between demes, and migration rate (Flanagan & Jones, 2017). Basing management decisions on such analyses in isolation would be unadvisable. Alternatively, when true adaptive loci are unavailable, patterns described using LUPS can suggest population dynamics that go unrecognized by neutral loci, such as sensitivity to external stressors that management needs to account for or control (Vitalis, Dawson, & Boursot, 2001).

In response to these various limitations in the literature, we sampled 188 individuals from across the western and central Pacific Ocean (WCPO) in units that control for temporal and spatial variation, considering both neutral loci and LUPS. Our comparisons, while still not comprehensive, directly assess spatial and temporal population structure at neutral and adaptive loci in South Pacific albacore.

## 2 METHODS

**Figure 2:**
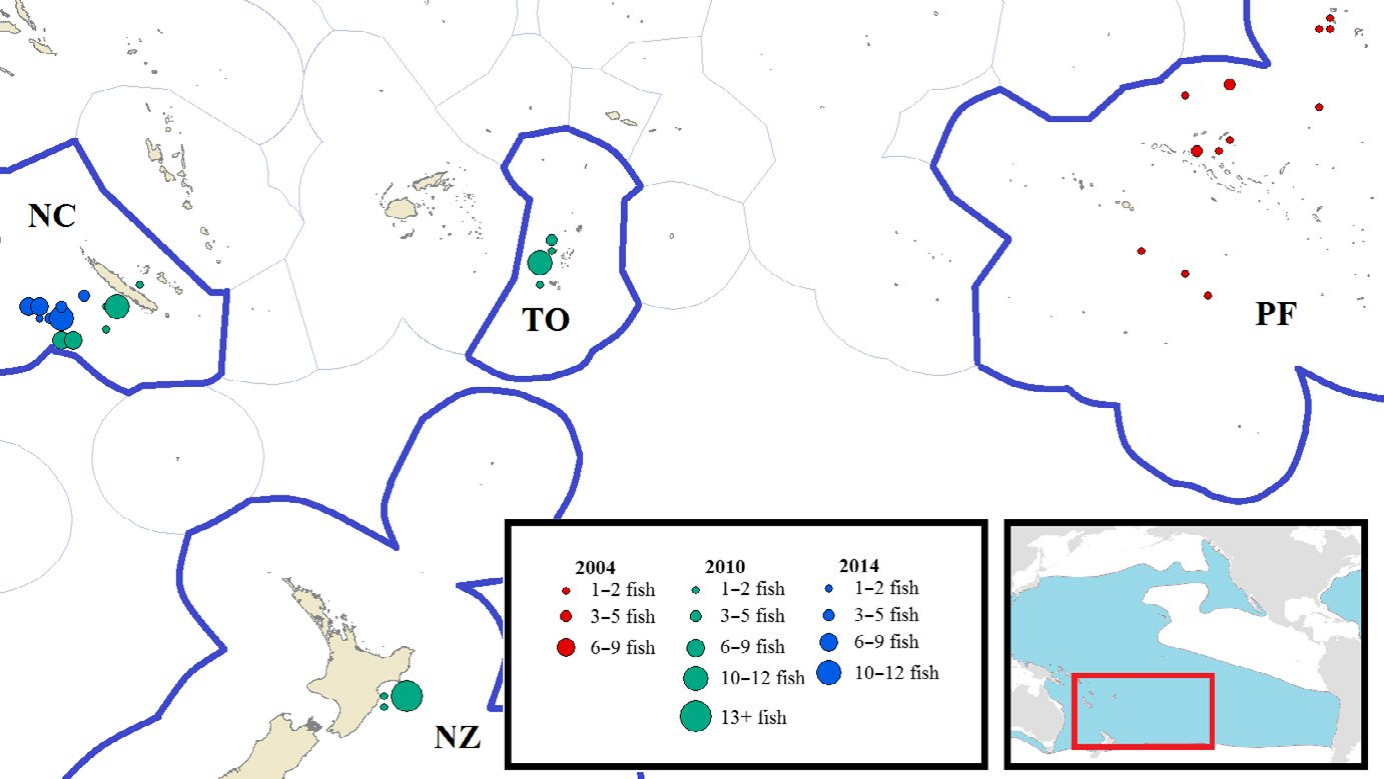
Map of sample sight locations. Size of circle represents the number of fish caught inside blue line of each country's EEZ boundaries. Inset: albacore range in the Pacific, from http://www.fao.org/figis/geoserver/factsheets/species.html

Fish specimens were selected for analysis from tissue samples archived in a tissue bank collection managed by the Pacific Community (SPC) under the auspices of the Western and Central Pacific Fisheries Commission (WCPFC), in Noumea, New Caledonia. Tissue samples consisted of muscle plug biopsies taken by observers on fishing vessels and by scientists during research cruises. Tissue samples within the archive reflect an opportunistic sampling distribution, from which specimens for this study were selected based on catch location and date, such that all samples in a grouping were collected within 6 weeks of each other, and within an area of 22,000 km^2^ (radius of 85 km). The exception is the population sample collected in French Polynesia in 2004 (PF04), which included all samples from that year and exclusive economic zone (EEZ), regardless of location or season, in order to attain an adequate sample size. Population samples were selected to represent groups from New Caledonia in 2010 and 2014 (NC10 and NC14, respectively), New Zealand and Tonga also in 2010 (NZ10 and TO10, respectively), and the aforementioned group PF04 from French Polynesia in 2004. NC10 and NC14 were chosen to provide a temporal comparison that controls for both location and season. Similarly, NC10, NZ10, and TO10 consist of specimens collected within the same two‐month period, in order to produce a concisely controlled spatial comparison. All samples were accompanied by metadata including catch location, date, and catch event number, and fish size (Table S1). A total of 188 individuals from five geographic locations were analyzed, representing four countries and spanning 10 years (Figure 2).

DNA extraction and sequencing were conducted by Diversity Arrays Technology (DArT PL). Its patented next‐generation sequencing protocol, DArTseq, is a cost‐effective option for generating high‐quality, high‐throughput SNP datasets for nonmodel species. Although some steps are proprietary knowledge, a description of the DArTseq protocol is available in Kilian et al., (2012), Sansaloni et al. (2011), and Ren et al. (2015). Following automated DNA extraction, samples were digested using *Pst*I and *Sph*I restriction enzymes. Methylation‐sensitive enzymes were chosen to avoid highly repetitive, methylated genomic regions that are minimally informative and tend to carry elevated risk of misinterpreting paralogs as a single locus during marker calling. Specialized adaptors were ligated to digested DNA. Both *Pst*I and *Sph*I adapters included a PCR primer sequence and Illumina flowcell attachment sequence, and the *Pst*I adaptor also included a unique, varying length barcode sequence for sample recognition within pooled libraries. PCR only amplified fragments capped with both adaptors, using the following protocol: 1‐min denaturation at 94°C, 30 cycles of 20 s at 94°C, 30 s and 58°C and 45 s at 72°C, and a final extension step of 7 min at 72°C. Libraries were then further amplified using bridge PCR on the Illumina HiSeq 2500 platform and sequenced on the same platform. The resulting data were submitted to an in‐house software, DArTsoft, which interprets sequences from images of fluorescence taken during Illumina sequencing and produces FASTQ files. Files were quality controlled for sequences with 90% confidence at 50% of bases and split by barcode into individual specimens. Sequences were aligned de novo. A separate algorithm, DArTsoft14, called SNPs and further quality‐filtered for singletons and other suspected sequencing errors. The final output produced by DArT was a genotype report of all identified SNPs, their global call rate, polymorphic information content, and their codominant status in each sequenced specimen.

The returned dataset of 27,295 SNPs was further filtered for locus quality. Loci were first culled by removing all but one SNP per sequenced DNA fragment. Remaining loci were selected based on a 99% call rate, a minimum read depth of 7×, and 5% minor allele frequency. *F*_ST_ outlier analyses were conducted with LOSITAN v. 2.1 using the prior odds for neutral model and a 10% false discovery rate. Individuals were submitted to LOSITAN in their five original sample groups. Next, loci were extracted that showed deviation from Hardy–Weinberg Equilibrium (HWE) across all populations. HWE tests whether loci occur at frequencies that deviate from selectively neutral assumptions and were analyzed using Arlequin v. 3.5.2.1 (Excoffier & Lischer, 2010). The large number of loci used in this study complicates HWE testing significance because a standard *p*‐value of .05 would prompt the unnecessary discard of 100 or more informative loci through Type I error if the threshold for significance was not adjusted accordingly (Waples & Allendorf, 2015). Because Alrequin does not calculate a *p*‐value small enough to reflect accurate correction for multiple testing, we used the most sensitive available *p*‐value threshold of .0001. HWE results were also filtered for loci with a maximum observed heterozygosity of 0.5, as an independent control for the potential merging of paralogous loci in the DArTseq pipeline. Finally, pairwise linkage disequilibrium was assessed using PLINK2 (Chang et al., 2015) and a threshold of 70% linkage between loci. All filtering steps were first conducted on all five population samples and then repeated using just NC10 and NC14 to provide a maximally informative dataset for analyses that controlled for spatial distribution of samples when exploring temporal variation in population structure. Raw datasets are publicly available from Open Science Framework under Digital Object Identifier 10.17605/OSF.IO/QD7BW.

The final datasets were used to calculate pairwise *F*_ST_ in Arlequin v 3.5.2.1 (Excoffier & Lischer, 2010) using 10,000 permutations for significance. The number of distinct genetic clusters, *k*, was selected in ADMIXTURE v. 1.3.0 (Alexander, Shringarpure, Novembre, & Lange, 2015) by coercing the given samples into between 1 and 10 clusters and comparing the resulting amount of cross‐validation error (CV) among the different *k* values. The *k* with the lowest associated CV was selected. ADMIXTURE outputs from the recommended *k* value were then visualized using the “barplot ()” command in R v. 3.3.1 and cross‐checked using the R package stockr and the command “stockSTRUCTURE.” Another visual assessment of each dataset was produced using a discriminant analysis of principal components (DAPC) in the R package *adegenet* after alpha optimization (via R commands “dapc ()” and “a.score.optim ()”). The *adegenet* package was also used to independently recommend the number of genetic clusters based on the Bayesian information criterion (BIC), although this always concurred with interpretations of ADMIXTURE. Genodive v. 2.0b27 (Meirmans & van Tienderen, 2004) was used for an independent recommendation of population assignment using the original five sample groups and an analysis of molecular variance (AMOVA).

The neutral dataset was also used to calculate adjusted expected and observed heterozygosities (*H*_n.b._ and *H*_o_), and the inbreeding coefficient *F*_IS_ (at 1,000 permutations), using GENETIX v 4.05 (Belkir, Borsa, Chikhi, Raufaste, & Bonhomme, 2004). These values are only informative under assumptions that include neutrality and therefore were not calculated for the LUPS dataset. Basic statistical comparisons were made using the chi‐square test for homogeneity in R, using the core command “chisq.test.”

## 3 RESULTS

Sequencing of 188 individuals from five population samples using DArTseq identified 27,295 loci after Diversity Array Technology's in‐house quality filtering protocols using the DArTtoolbox. Forty‐one individuals were discarded from reporting due to their inability to produce adequate quality sequencing data, including 22 from TO10, six from NC10, five from NC14 and PG04, and three from NZ10. Secondary quality filtering of the remaining specimens from all five population samples produced a neutral dataset of 1,837 loci and a LUPS dataset of 89 loci. When the two New Caledonia samples, NC10 and NC14, were filtered independent of other samples, 1,925 neutral and 66 purportedly selected loci were identified (Table 1).

**Table 1:** Number of loci remaining after each quality filtering step for datasets using neutral loci (NL) and loci under potential selection (LUPS)

Filtering step	All population samples		New Caledonia samples	
	NL	LUPS	NL	LUPS
Initial	27,295	27,295	27,295	27,295
Duplicates on contig	18,900	18,900	18,900	18,900
Call rate (99%)	6,351	6,351	6,351	6,351
Read depth (7x)	5,624	5,624	5,624	5,624
MAF (5%)	2,144	2,144	2,230	2,230
*F*_ST_ outliers (CI 10%)	2,011	**89**	2,163	**66**
HWE (*p* < .0001)	2,011	–	2,163	–
*H*_O_ (<.5)	1,837	–	1,925	–
LD (70%)	**1,837**	–	**1,925**	–

Note

Bolded values are the final number of loci per dataset.

Neutral global analyses demonstrated underwhelming evidence of population structure. As the most fundamental measures of population structure, diversity assessments were unremarkable. Adjusted expected heterozygosities all fell between 0.24 and 0.26, while observed heterozygosities ranged from 0.22 to 0.26 (Table 2). No chi‐square comparisons of the expected and observed values per population samples were significantly different (*χ* ^2^ = .0004, *p* = 1). The inbreeding coefficient *F*_IS_ ranged from −.001 (NC10) to .07 (TO10) (Table 2), none of which are statistically significantly different from 0 using a chi‐square test (*χ* ^2^ = .0019, *p* = 1).

**Table 2:** Measures of diversity for each population sample, including the inbreeding coefficient *F*_IS_, adjusted expected heterozygosity *H*_n.b._, observed heterozygosity *H*_o_, and sample size *n*

	*H*_o_	*H*_n.b._	*F*_IS_	*n*
NC10	.2554 (±.145)	.2551 (±.131)	−.0015	34
NC14	.2474 (±.138)	.2529 (±.131)	.0222	35
NZ10	.2371 (±.131)	.2475 (±.130)	.0427	39
PF04	.2480 (±.153)	.2527 (±.138)	.0192	21
TO10	.2244 (±.145)	.2420 (±.143)	.0748	18

**Figure 3:**
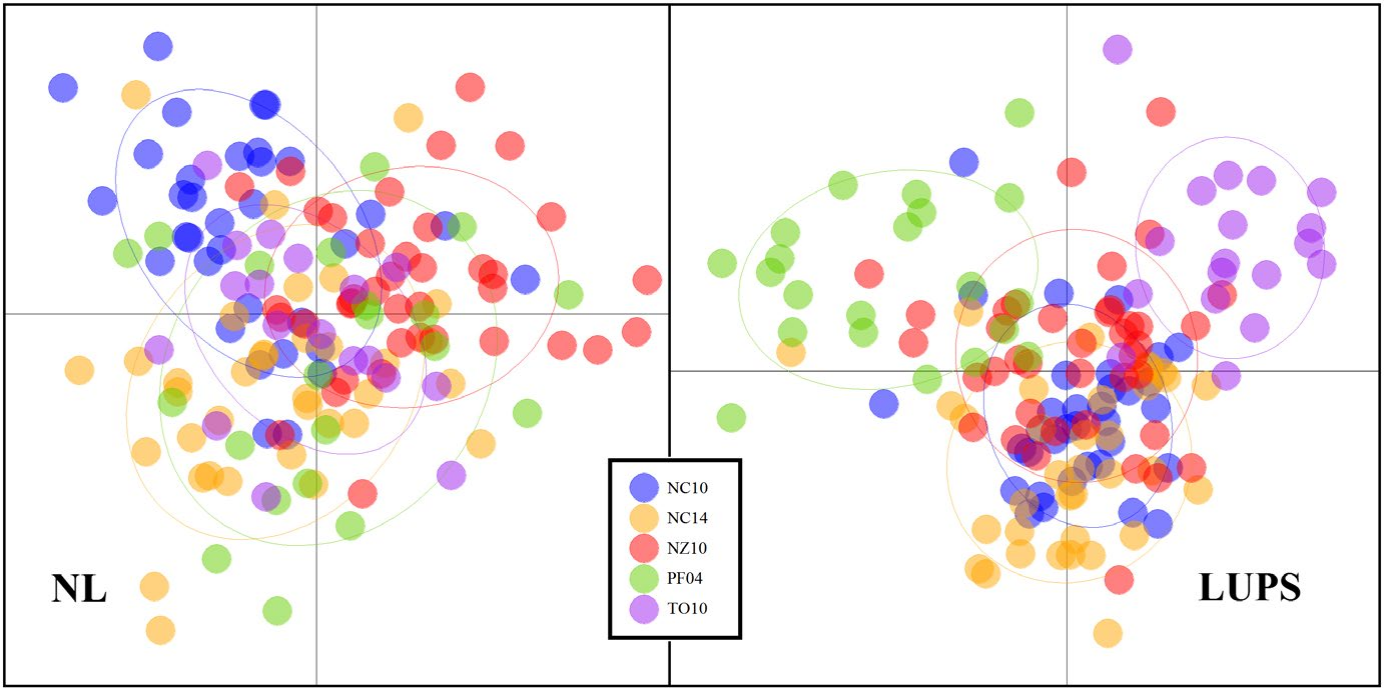
DAPC calculated for all individuals. Analysis of neutral loci (left panel) used 24 principal components and 4 *df*; analyses of loci under potential selection (right panel) used eight principal components and 4 *df*

Comparative assessments using neutral loci were likewise rarely significant. Of 10 possible pairwise *F*_ST_ values, only two are statistically significant after Bonferroni correction: NZ10 with either NC10 or NC14 (Table 3). However, those values are extremely low at .003 (*p* = .0004 and .0002, respectively). Likewise, ADMIXTURE software identified *k* = 1, indicating only one genetic cluster was present among all samples. An alpha‐optimized DAPC using 24 principal components and 4 *df* similarly showed no clear distinction between population samples (Figure 3) and related interpretations of BIC recommended the presence of one genetic cluster. Concurrently, Genodive population assignments accurately identified between 0% (TO10) and 67% (NZ10) of individuals, with an average of 32%. An AMOVA placed 97.1% of variation within individuals and only 0.2% between population samples (*p* = .001).

**Table 3:** Pairwise *F*_ST_ values (below diagonal) and associated *p* values (above diagonal) between population samples using neutral loci

	NC10	NC14	NZ10	PF04	TO10
NC10	–	.0538	**.0004**	.0812	.2111
NC14	.0016	–	**.0002**	.1979	.3833
NZ10	**.0027**	**.0031**	–	.0638	.4563
PF04	.0018	.0014	.0024	–	.4695
TO10	.0014	.0011	.0011	.0011	–

Note

Bold indicates values that are statistically significant.

**Figure 4:**
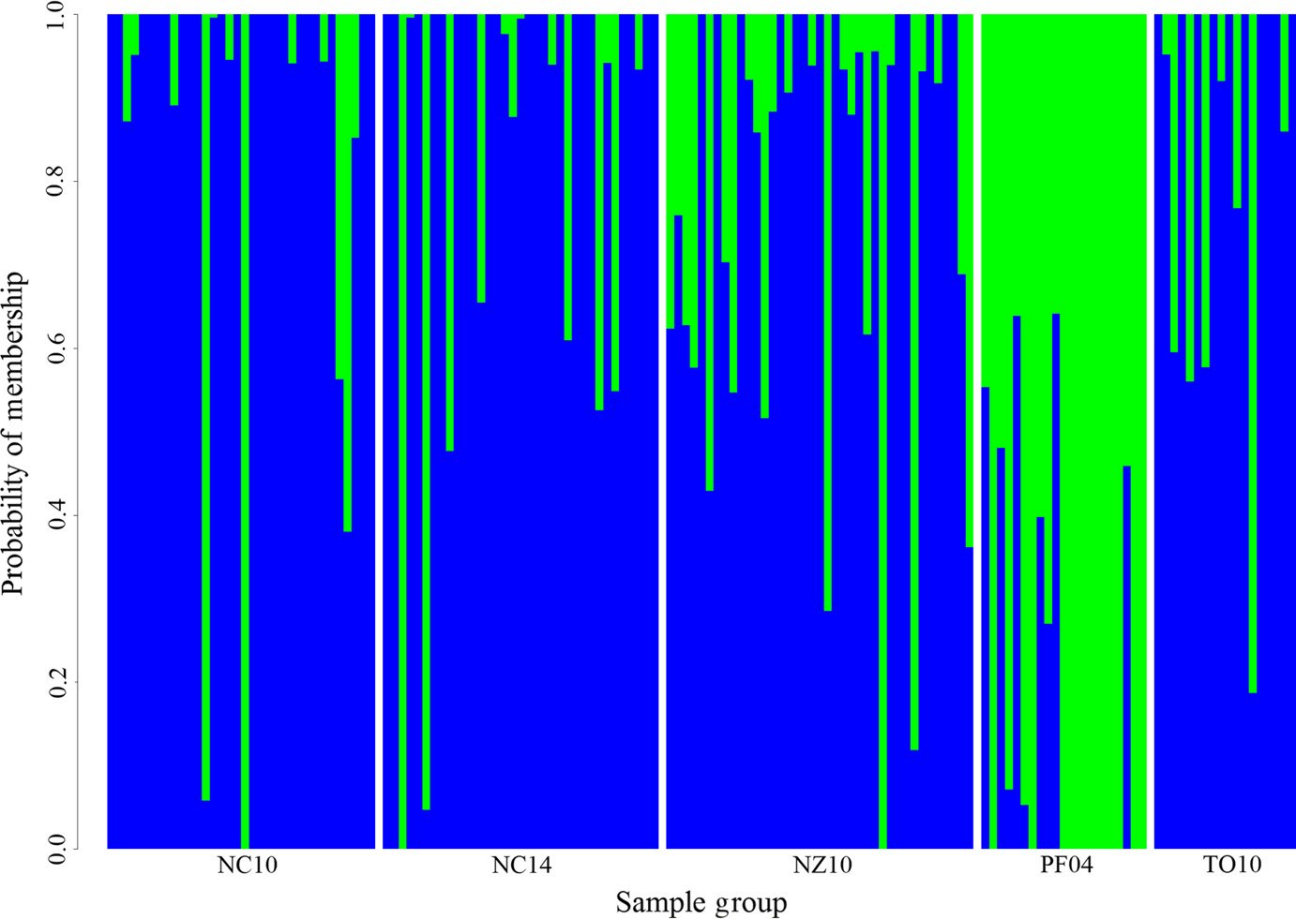
Probability of population placement assuming there are two true populations among the observed samples (*k* = 2), as recommended by ADMIXTURE for the LUPS dataset

By comparison, analyses using LUPS provided evidence of population structure. Pairwise *F*_ST_ values were all statistically significant and ranged from .016 (NC10 vs. NZ10, *p* = .001) to .176 (PF04 vs. TO10, *p* < .00001) (Table 4). All pairwise *F*_ST_ values above 0.1 included PF04, and there is a difference of only .005 between values produced by the three lowest values, all of which combined NC10, NC14, and NZ10. ADMIXTURE identified *k* = 2 and separated 30 individuals into the second group, including 18 of 21 individuals in PF04 (Figure 4). An alpha‐optimized DAPC using eight principal components and 4 *df* produced similar results, with limited overlap of samples from PF04 with those from NC10, NC14, and NZ10. TO10 showed a similar degree of separation as PF04 (Figure 3). Improved distinction between sample groups also reflected in Genodive population assignments, where the global average rose to 70%, ranging from 56% (NZ10) to 76% (PF04). Likewise, an AMOVA of LUPS identified more variation between groups than the same analysis using NL data, with 90% of variation within individuals, 7% allocated between population samples (*p* = .001), and another 3% among individuals within populations (*p* = .003).

**Table 4:** Pairwise *F*_ST_ values (below diagonal) and associated *p* values (above diagonal) between population samples using loci under potential selection

	NC10	NC14	NZ10	PF04	TO10
NC10	–	**.0004**	**.0012**	**.0000**	**.0000**
NC14	**.0164**	–	**.0000**	**.0000**	**.0000**
NZ10	**.0157**	**.0203**	–	**.0000**	**.0000**
PF04	**.1609**	**.1558**	**.1278**	–	**.0000**
TO10	**.0516**	**.0630**	**.0465**	**.1761**	–

Note

Bold indicates values that are statistically significant.

**Figure 5:**
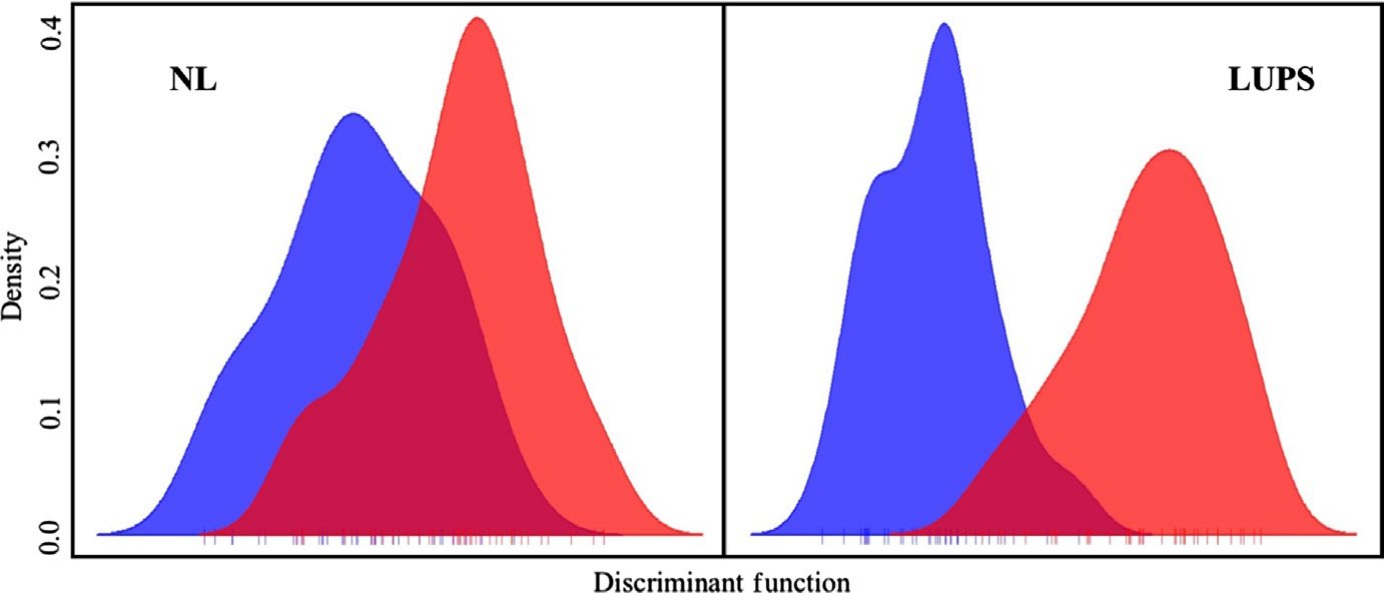
DAPC calculated for two sample groups from New Caledonia, NC10 and NC14. Neutral analyses (left panel) use 23 principal components and 1 *df*. Loci under potential selection (right panel) use one principal component and 1 *df*

Using the New Caledonia‐specific dataset, analysis of neutral loci again provided evidence of temporal stability in the allele frequencies. Pairwise *F*_ST_ values were not significant and uninformative, at .0003 with *p*‐value .76. ADMIXTURE recommended *k* = 1 and a DAPC using 23 principal components and one degree of freedom showed major overlap between sample groups (Figure 5). An AMOVA produced similar results to the global neutral dataset, with 97.7% of variation found within individuals (*p* = .001), and population assignment was only correct 41% of the time. The evidence produced by LUPS is more complex. The pairwise *F*_ST_ value was comparable to those produced by the global dataset (.08, *p* < .000), as were an AMOVA (90% variability within the individual and 8% between populations, *p* = .001). Population assignment was even more accurate than that of global analyses, with all but one individual correctly assigned (98% accuracy). However, although a DAPC visibly separated the two groups using one principal component (Figure 5), the recommended number of genetic clusters from ADMIXTURE and *adegenet* both dropped to 1.

## 4 DISCUSSION

Based on the current results, we infer that the central and western South Pacific supports a single, genetically healthy population of albacore tuna that is notably substructured in some genetic regions, potentially due to environmental factors that can produce local adaptation. Observations of neutral genetic diversity and robustness all follow precedent set by other studies of albacore and various tuna species and do not indicate any need for conservation concerns. Assessments using LUPS demonstrate genetic differentiation between population samples, although the overlapping impact of time, distance, and cohort sampling strategy cannot be definitively isolated using the current sample distribution.

### 4.1 Panmixia at neutral loci

None of the analyses of this study that used neutral loci identified any significant population structure. Homozygosity values mirror those of other pelagic species using similar study designs including yellowfin tuna (*Thunnus albacares,* average *H*_e_ = .24, average *H*_o_ = .26 using DArTseq‐produced SNPs; Grewe et al., 2015) and dolphinfish (*Coryphaena hippurus,* average *H*_e_ = .25, average *H*_o_ = .25 using RADseq‐produced SNPs; Maroso, Franch, Dalla Rovere, Arculeo, & Bargelloni, 2016), and only slightly below that of swordfish (*Xiphias gladius,* average *H*_e_ = .29 using HRMA methods; Lu, Smith, Hinton, & Alvarado Bremer, 2016). None of the listed species are mentioned on the IUCN Red List for conservation (Collette, Acer, et al., 2011; Collette, Acero, Amorim, Bizsel, et al., 2011; Collette, Acero, Amorim, Boustany, et al., 2011), or highlighted for concern in Western and Central Pacific Fisheries Commission stock assessments (ISC Billfish WG, 2018; Tremblay‐Boyer, McKechnie, Pilling, & Hampton, 2017), implying that albacore should be a similarly robust species. This study's comparative values of *H*_o_ and *H*_n.b._, or adjusted *H*_e_, along with the inbreeding coefficient *F*_IS_, likewise do not indicate any concern for the genetic health of albacore in the WCPO. The lack of difference between expected and observed heterozygosities indicates that the assumptions behind Hardy–Weinberg Equilibrium hold true for this population: it is very large, temporally stable, and panmictic. Likewise, *F*_IS_ values are not significantly different from 0, again providing no evidence of limited breeding option or, by extension, population substructuring.

Comparative analyses such as ADMIXTURE, DAPCs, and pairwise *F*_ST_ also provided no evidence to challenge panmixia at neutral loci within the sample range. Neither calculations of *k* nor visuals produced by a DAPC offer any hint of population differentiation among our sample groups. Only two pairwise *F*_ST_ calculations are statistically significant, between NZ10 and either NC10 and NC14, but the small absolute values of the comparisons actually represent a 0.3% difference between groups and may not indicate biological significance. By comparison, Knutsen et al. (2011) addressed the same question for coastal Atlantic cod and defended the biological significance of what he called “very small” *F*_ST_ values that average .0037 or 190% of the value in question here. If the same *F*_ST_ estimator was used in both studies, the discrepancy would increase to 250%. However, there are numerous noncomparable elements between studies. Most fundamentally, Knutsen et al. (2011) used much more thorough sampling, with 1,300 individuals collected across 13 sample sites compared with 147 individuals across five samples sites in the current study. In reality, species life history, extent of population structure, specific types of analyses, and number and polymorphic richness of loci all provide necessary context to determine the number of samples and sample groups necessary to identify population structure, with different conditions meriting from 10 to 200 individuals per sample group (Hoban, Gaggiotti, & Bertorelle, 2013; Pudovkin, Zhdanova, & Hedgecock, 2010). Although there is no simple rule about sample size, incorporating more samples invariable reduces the risk of sampling error (Nei, 1978), lending much greater confidence to the relevance of shallow population structure demonstrated in Knusten et al. (2011) than the current study. Furthermore, effective population size (*N*_e_) is also much larger in cod than those characteristically found in mobile pelagic species (Knutsen et al., 2011), suggesting that coastal Atlantic cod do not follow the same sweepstakes reproduction model that is believed to produce extremely small *N*_e_ values in pelagic species such as tuna (Waples, 2016). Again, demonstration of biological relevance of low *F*_ST_ values in a low‐dispersal coastal fish using different sampling patterns cannot directly justify the significance of similar results in highly mobile pelagic species such as tuna. Finally, AMOVA results from Knutsen et al. (2011) allocated more than twice as much variance between populations as the current analyses for albacore given similar *F*_ST_ values, providing better cross‐validation for that study's potentially relevant pairwise *F*_ST_ values.

Our observations of neutral panmixia within the WCPO follow a long tradition of similar conclusions. Differentiation between ocean basins is well established; very few studies fail to find differentiation between water bodies, and those that do are often poorly sampled (Graves & Dizon, 1989), or use sample sites that are technically in different bodies of water, but in fact are very close to the border (Pujolar, Roldan, & Pla, 2003). However, many studies that successfully describe separation of albacore from separate oceans fail to sense differentiation between Pacific samples. These include Davies et al. (2011), which sampled two locations in the southwest Pacific; Albaina et al. (2013), which used three samples from the southwest, southeast, and North Pacific; Laconcha et al. (2015), which similarly sampled the southwest, south‐central, and northeast Pacific; and Chow and Ushiama (1995) that took 10 samples from all across the ocean basin.

In contrast, tuna Regional Fishery Management Organisations (tRFMO) currently divide both the Atlantic and Pacific Oceans into two stocks, separated by hemisphere. Molecular and tagging studies support such organization in the Atlantic; blood group antigens associated the South Atlantic with the Indian Ocean but differentiated the North Atlantic (Arrizabalaga et al., 2004), and tagging studies demonstrated absolutely no movement between the North and South Atlantic (Arrizabalaga et al., 2002). However, albacore's distribution in the Pacific is different, with no presence in the cold tongue of the equatorial East Pacific, yet historically acknowledged presence at the same latitudes in the West Pacific Warm Pool (Collette, Acero, Amorim, et al., 2011). Albacore are known to remain within a body of water while migrating, rather than cross thermoclines (FAO, 1985), which in most oceans would deter movement through equatorial waters. The unique oceanography of the western Pacific could create a corridor of habitability across the equator, providing at least one mechanism to homogenize the two hemispheres.

### 4.2 Evidence of population genetic structure at potentially adaptive loci

The current study is one of the relatively few that identifies and conducts analyses on potentially adaptive loci in albacore. As with comparable studies, our assessments incorporating LUPS detected much greater potential for structure than those using neutral loci; pairwise *F*_ST_, DAPC, and estimations of *k* all differentiated PF04 from the rest of the population samples, with secondary distinction of TO10. The elevated success in sensing differentiation is similar to that of Laconcha et al. (2015), which analyzed albacore using 58 neutral SNPs and was unable to confidently distinguish between any sample groups on a global scale, but added 17 purportedly adaptive loci and successfully differentiated the Mediterranean, Atlantic, Pacific, and to a lesser degree the Indian Oceans. Montes et al. (2012) likewise used eight neutral microsatellites and could only distinguish Mediterranean samples from a group of the three ocean basins, but could further distinguish Atlantic and Indian samples from Pacific groups when a single LUPS was added. Montes et al. (2012) also found notable divisions between samples from the southwest Pacific and both the northwest and southeast Pacific, although they were not statistically significant.

The differentiation observed in the current analyses was much greater than those reported for albacore by either Montes et al. (2012) or Laconcha et al. (2015). Our lowest pairwise *F*_ST_ value was produced by a comparison of NC10 and NZ10, at .016. This is greater than Laconcha et al.’s comparison between samples from the North Atlantic and Indian Oceans, and on par with Montes et al.’s comparisons between Pacific and Mediterranean samples. Our most divergent comparisons all include PF04 and are a magnitude higher than any global value produced by either Montes et al. or Laconcha et al. This could partly be because both studies acknowledged the presence of LUPS but retained them within a larger, predominantly neutral dataset, while the present study specifically isolated LUPS from the neutral dataset and therefore did not experience any dilution of the adaptive structure signal. It could also be a question of sample selection.

TO10 and PF04 were both distinctly different in their specimen distribution from other sample groups. As was highlighted with neutral analyses, TO10 included a regrettably small sample size due to difficulties during sequencing, probably because of inadequate sample preservation. The accuracy of its representation of the larger population must therefore be considered with caution and likewise any observations of population genetic differentiation based on it. The case of PF04 was more complicated. Collected in French Polynesia in 2004, it was geographically and temporally the outlier of our dataset. PF04 is not directly comparable to any other population sample, whether standardized by location, season, or year. Specimens that make up PF04 were opportunistically collected over a large area and over the course of 10 months, whereas other sample groups were from a very concise area and taken within six weeks. PF04 was also located pointedly more north than other groups, with an average latitude of 14°S, compared to an average of 22°S between samples collected in the next three most northerly groups from Tonga and New Caledonia. It therefore cannot be determined which of these deviations was most responsible for the large differentiation values. However, even if genomic distinctiveness was primarily a result of sampling abnormalities, rather than a genuine difference between population samples, that observation still carries implications for the presence of cryptic temporal substructure at a single location, presumably based on seasonal migrations. In addition, given the still significant differentiation between much closer and more controlled comparisons, the extrapolation to PF04 was feasible, and not under suspicion of being a complete artifact.

Our observations are unique in the strength and ubiquity of the demonstrations of population genetic differentiation within the South Pacific. However, other forms of population observations have a long tradition of similar conclusions. Morphological comparisons of features including overall size and rate of maturity, and gonad‐to‐overall size ratio, have demonstrated significant differences between latitudes in the South Pacific. Adult albacore of the same sex and age, as assessed by otolith rings, are on average 6 cm smaller near New Caledonia than around Samoa and the Cook Islands (Harley, Peter, Nicol, Hampton, & Brouwer, 2015; Williams et al., 2012). Similarly, albacore in the eastern South Pacific have larger gonads relative to their somatic weight than specimens in the western Pacific (Farley et al., 2013) and reach first maturity at a slightly smaller size (Farley et al., 2014). Growth and reproductivity are often influenced by factors including oxygen concentration, temperature, and prey availability (Murua et al., 2017); however, differences in absolute size reported by Williams and Terawasi (2013) did not correlate with gradients in any abiotic factor. It is possible that there is a genetic element convoluting the relationship. All of these observations also concur with our analyses that distinguish PF04, and to a lesser degree TO10, from western Pacific samples.

### 4.3 Difficulties in describing a convoluted life history

The overarching picture of neutral panmixia and strong local adaptation that we propose is a useful marriage of previously conflicting genetic and observational reports. However, it still does not fully capture the complex, migratory life history of this species. Satellite tagging studies have demonstrated incomplete seasonal migration habits in the northeastern Pacific, wherein juveniles follow any of five basic routes between the coast and wintering waters in the central Pacific, and can vary their route between years (Childers et al., 2011). Likewise, strontium to calcium ratios in the otoliths of North Atlantic juveniles indicated that specimens from adjacent feeding grounds started in the same nursery waters but took different migratory routes and experienced different salinity and temperature regimes, which are environmental factors that could influence local adaptation (Duncan et al., 2018; Fraile et al., 2016). All of these studies established a single spawning ground per hemisphere per ocean, from which young albacore disperse unpredictably. No genetic study to date has successfully isolated migrating juveniles based on route to assess whether the various groups are genetically as well as morphologically distinct, or whether the differentiation persists over years.

Similarly, tuna species are known to be highly sensitive to the El Niño Southern Oscillation (ENSO) cycle in the Pacific, with skipjack and yellowfin both showing predictable changes in distribution and recruitment during ENSO events (Lehodey, Bertignac, Hampton, Lewis, & Picaut, 1997; Nicol et al., 2014). Albacore are no different; CPUE data indicated a reduced recruitment during El Nino events that is strongest in the western Pacific but evident at all longitudes, with a lag time that increases toward the east up to two years after the event (Lu, Lee, & Liao, 1998; Singh, Sakuramoto, & Suzuki, 2015). Furthermore, because of albacore's longer life span and more seasonal reproductive activity compared with tropical tuna species, downstream impacts of an ENSO event can be felt for up to 8 years, depending on location of observation (Lu et al., 1998). Any environmental event that significantly reduces the size of a cohort will also carry implications for the population genetic health and diversity of cohorts produced during that time through the increased influence of genetic drift and will change the selective pressure on various alleles and genomic regions. But, again, no genomic study has been designed to isolate the impact of ENSO events on albacore population genetic structure.

It is evident that Pacific albacore life history includes numerous forms of population structure that have not yet been assessed from a genetic basis. The success of the current analyses at describing potentially adaptive differentiation within the Pacific, combined with the environmental basis of many externally identified morphological differences, encourages continued exploration of the distribution in space and time of the adaptive genetic diversity of albacore and of the implications for fisheries. Especially, considering the ever‐decreasing trend in costs of genome‐wide SNP sequencing (Therkildsen & Palumbi, 2017), continued exploration of the albacore genome for regions under potential selective pressure should remain a priority in the near future.

## 5 CONCLUSION

In sum, we have demonstrated panmixia in albacore tuna in the WCPO using 1,837 neutral SNP loci and indications of population genetic structure at potentially adaptive regions of the genome using 89 LUPS. Well‐controlled specimen selection in four of five population samples to establish that, using loci that do not comply with neutral allele distribution, differentiation occurs between geographically distant locations sampled at the same time, and within one location when sampled in the same season but different years. Although one group, TO10, is undersampled and potentially poorly represented, the pattern of differentiation persists with or without the suspect data.

The differentiation between any of these samples and the fifth group, PF04, is a magnitude higher than the already notable separation among the original four. It is unclear whether the dramatic genetic differentiation stems more from the geographic distance between sample groups, the larger number of intervening years, or the distinctly more dispersed specimen selection, which includes individuals caught in both the tropics and subtropics, and over a span of 10 months. Whatever combination of these factors proves to be relevant, the fundamental deviation from panmixia deserves further exploration.

Without understanding the environmental basis and interaction that prompts adaptive differentiation within the WCPO, it is not yet appropriate to recommend changes to current management practices. However, further exploration of the present observations must be prioritized in order to identify the driving factors, establish the relevance to long‐standing observations of morphological differentiation, and potentially update management assumptions to reflect this new reality.

## CONFLICT OF INTEREST

The authors identify no competing interests.

## AUTHOR CONTRIBUTIONS

CR obtained funding from the University of the South Pacific. CR and GA designed the study. GA performed the research, analyzed the data, and wrote the manuscript. JH and NS, as agents of the Pacific Community (SPC), critically assessed the results from a fisheries perspective. SPC was also responsible for sample collection, facilitated sample access, and prepared Figure 2. All authors contributed to the preparation of the final manuscript.

## Data Availability

Raw SNP datasets are publicly available from Open Science Framework under Digital Object Identifier 10.17605/OSF.IO/QD7BW.
