# Supplementary material for: Indications of strong adaptive population genetic structure in albacore tuna (Thunnus alalunga) in the southwest and central Pacific Ocean
Source: Ecol Evol. 2019 Aug 27;9(18):10354–64. doi: 10.1002/ece3.5554 (PMC6787800; doi:10.1002/ece3.5554)
Supplement: Supplementary file 1 [file ECE3-9-10354-s001.docx]

**Supplementary Table 1** Metadata for all specimens including catch location, date, and catch event number, and fish size and sex.

| Population Sample | ID | Date | Latitude | Longitude | Length (mm) | Weight (calculated, gr) | Sex |
| --- | --- | --- | --- | --- | --- | --- | --- |
| NC14 | 7 | 20-Jun-14 | -22.67 | 162.91 | 880 | 10378 | F |
| NC14 | 8 | 20-Jun-14 | -22.67 | 162.91 | 920 | 11806 | F |
| NC14 | 9 | 20-Jun-14 | -22.67 | 162.91 | 860 | 9708 | F |
| NC14 | 10 | 20-Jun-14 | -22.67 | 162.91 | 860 | 9708 | F |
| NC14 | 11 | 20-Jun-14 | -22.67 | 162.91 | 900 | 11077 | F |
| NC14 | 13 | 21-Jun-14 | -22.63 | 162.85 | 810 | 8159 | F |
| NC14 | 14 | 21-Jun-14 | -22.63 | 162.85 | 940 | 12566 | M |
| NC14 | 15 | 23-Jun-14 | -22.56 | 162.77 | 850 | 9384 | F |
| NC14 | 17 | 23-Jun-14 | -22.56 | 162.77 | 860 | 9708 | M |
| NC14 | 18 | 23-Jun-14 | -22.56 | 162.77 | 910 | 11438 | F |
| NC14 | 19 | 23-Jun-14 | -22.56 | 162.77 | 950 | 12958 | M |
| NC14 | 20 | 24-Jun-14 | -22.38 | 162.67 | 920 | 11806 | M |
| NC14 | 21 | 24-Jun-14 | -22.38 | 162.67 | 920 | 11806 | M |
| NC14 | 22 | 24-Jun-14 | -22.38 | 162.67 | 940 | 12566 | M |
| NC14 | 23 | 25-Jun-14 | -22.62 | 162.37 | 920 | 11806 | M |
| NC14 | 24 | 26-Jun-14 | -22.63 | 162.03 | 960 | 13358 | F |
| NC14 | 25 | 26-Jun-14 | -22.63 | 162.03 | 870 | 10039 | M |
| NC14 | 26 | 27-Jun-14 | -22.44 | 161.92 | 850 | 9384 | F |
| NC14 | 27 | 27-Jun-14 | -22.44 | 161.92 | 960 | 13358 | M |
| NC14 | 28 | 27-Jun-14 | -22.44 | 161.92 | 850 | 9384 | M |
| NC14 | 29 | 27-Jun-14 | -22.44 | 161.92 | 880 | 10378 | M |
| NC14 | 30 | 05-Jul-14 | -21.80 | 163.57 | 860 | 9708 | F |
| NC14 | 31 | 05-Jul-14 | -21.80 | 163.57 | 930 | 12182 | M |
| NC14 | 32 | 06-Jul-14 | -21.60 | 163.57 | 870 | 10039 | F |
| NC14 | 35 | 27-Jul-14 | -22.32 | 161.53 | 940 | 12566 | M |
| NC14 | 36 | 28-Jul-14 | -22.14 | 161.68 | 900 | 11077 | F |
| NC14 | 37 | 28-Jul-14 | -22.14 | 161.68 | 930 | 12182 | M |
| NC14 | 38 | 29-Jul-14 | -22.34 | 161.38 | 770 | 7044 | M |
| NC14 | 39 | 29-Jul-14 | -22.34 | 161.38 | 930 | 12182 | M |
| NC14 | 40 | 29-Jul-14 | -22.34 | 161.38 | 810 | 8159 | M |
| NC14 | 41 | 29-Jul-14 | -22.34 | 161.38 | 970 | 13766 | M |
| NC14 | 42 | 29-Jul-14 | -22.34 | 161.38 | 930 | 12182 | M |
| NC14 | 43 | 29-Jul-14 | -22.34 | 161.38 | 920 | 11806 | M |
| NC14 | 44 | 29-Jul-14 | -22.34 | 161.38 | 900 | 11077 | F |
| NC14 | 45 | 30-Jul-14 | -22.53 | 161.56 | 1040 | 16850 | M |
| NC10 | 83 | 21-May-10 | -22.35 | 165.28 | 870 | 10039 | F |
| NC10 | 84 | 21-May-10 | -22.35 | 165.28 | 870 | 10039 | F |
| NC10 | 85 | 21-May-10 | -22.35 | 165.28 | 840 | 9067 | F |
| NC10 | 86 | 21-May-10 | -22.35 | 165.28 | 880 | 10378 | F |
| NC10 | 87 | 21-May-10 | -22.35 | 165.28 | 870 | 10039 | F |
| NC10 | 88 | 21-May-10 | -22.35 | 165.28 | 900 | 11077 | M |
| NC10 | 89 | 21-May-10 | -22.35 | 165.28 | 830 | 8758 | F |
| NC10 | 90 | 21-May-10 | -22.35 | 165.28 | 840 | 9067 | F |
| NC10 | 91 | 21-May-10 | -22.35 | 165.28 | 840 | 9067 | F |
| NC10 | 92 | 21-May-10 | -22.35 | 165.28 | 910 | 11438 | M |
| NC10 | 93 | 21-May-10 | -22.35 | 165.28 | 830 | 8758 | F |
| NC10 | 94 | 22-May-10 | -22.15 | 164.70 | 870 | 10039 | F |
| NC10 | 95 | 22-May-10 | -22.15 | 164.70 | 800 | 7870 | M |
| NC10 | 96 | 26-Jun-10 | -23.71 | 162.75 | 880 | 10378 | M |
| NC10 | 98 | 26-Jun-10 | -23.71 | 162.75 | 870 | 10039 | F |
| NC10 | 99 | 26-Jun-10 | -23.71 | 162.75 | 830 | 8758 | F |
| NC10 | 100 | 26-Jun-10 | -23.71 | 162.75 | 860 | 9708 | M |
| NC10 | 102 | 26-Jun-10 | -23.71 | 162.75 | 810 | 8159 | M |
| NC10 | 103 | 26-Jun-10 | -23.71 | 162.75 | 870 | 10039 | F |
| NC10 | 104 | 26-Jun-10 | -23.71 | 162.75 | 860 | 9708 | F |
| NC10 | 105 | 26-Jun-10 | -23.71 | 162.75 | 870 | 10039 | M |
| NC10 | 106 | 28-Jun-10 | -23.54 | 163.42 | 880 | 10378 | F |
| NC10 | 107 | 28-Jun-10 | -23.54 | 163.42 | 890 | 10723 | F |
| NC10 | 108 | 28-Jun-10 | -23.54 | 163.42 | 870 | 10039 | F |
| NC10 | 109 | 28-Jun-10 | -23.54 | 163.42 | 860 | 9708 | F |
| NC10 | 110 | 28-Jun-10 | -23.54 | 163.42 | 840 | 9067 | M |
| NC10 | 111 | 28-Jun-10 | -23.54 | 163.42 | 850 | 9384 | F |
| NC10 | 112 | 28-Jun-10 | -23.54 | 163.42 | 880 | 10378 | F |
| NC10 | 113 | 28-Jun-10 | -23.54 | 163.42 | 880 | 10378 | F |
| NC10 | 114 | 28-Jun-10 | -23.54 | 163.42 | 860 | 9708 | F |
| NC10 | 116 | 29-Jun-10 | -23.29 | 164.50 | 870 | 10039 | M |
| NC10 | 118 | 29-Jun-10 | -23.29 | 164.50 | 820 | 8455 | M |
| NC10 | 120 | 10-Jul-10 | -21.34 | 166.34 | 920 | 11806 | F |
| NC10 | 122 | 10-Jul-10 | -21.34 | 166.34 | 930 | 12182 | M |
| TO10 | 149 | 29-Jun-10 | -19.57 | -175.31 | 960 | 13358 | U |
| TO10 | 154 | 30-Jun-10 | -20.13 | -175.82 | 920 | 11806 | U |
| TO10 | 155 | 30-Jun-10 | -20.13 | -175.82 | 940 | 12566 | U |
| TO10 | 159 | 30-Jun-10 | -20.13 | -175.82 | 980 | 14181 | U |
| TO10 | 161 | 30-Jun-10 | -19.39 | -175.08 | 860 | 9708 | U |
| TO10 | 162 | 30-Jun-10 | -19.39 | -175.08 | 930 | 12182 | U |
| TO10 | 163 | 30-Jun-10 | -19.39 | -175.08 | 920 | 11806 | U |
| TO10 | 165 | 30-Jun-10 | -20.13 | -175.82 | 890 | 10723 | U |
| TO10 | 166 | 30-Jun-10 | -20.13 | -175.82 | 920 | 11806 | U |
| TO10 | 167 | 30-Jun-10 | -20.13 | -175.82 | 900 | 11077 | U |
| TO10 | 170 | 30-Jun-10 | -20.13 | -175.82 | 880 | 10378 | U |
| TO10 | 171 | 30-Jun-10 | -20.13 | -175.82 | 1010 | 15478 | U |
| TO10 | 173 | 30-Jun-10 | -20.13 | -175.82 | 910 | 11438 | U |
| TO10 | 174 | 30-Jun-10 | -20.13 | -175.82 | 1000 | 15037 | U |
| TO10 | 175 | 30-Jun-10 | -20.13 | -175.82 | 930 | 12182 | U |
| TO10 | 179 | 30-Jun-10 | -20.13 | -175.82 | 970 | 13766 | U |
| TO10 | 181 | 01-Jul-10 | -21.01 | -175.87 | 970 | 13766 | U |
| TO10 | 184 | 01-Jul-10 | -21.01 | -175.87 | 1000 | 15037 | U |
| PF04 | 195 | 22-Jan-04 | -12.54 | -146.54 | 980 | 14181 | F |
| PF04 | 197 | 26-Mar-04 | -13.08 | -140.63 | 1030 | 16384 | M |
| PF04 | 198 | 17-Sep-04 | -9.59 | -140.82 | 980 | 14181 | M |
| PF04 | 199 | 17-Sep-04 | -9.59 | -140.82 | 950 | 12958 | F |
| PF04 | 201 | 19-Sep-04 | -9.72 | -140.36 | 950 | 12958 | M |
| PF04 | 203 | 20-Sep-04 | -9.43 | -140.40 | 940 | 12566 | M |
| PF04 | 206 | 26-Aug-04 | -20.57 | -146.66 | 1070 | 18299 | M |
| PF04 | 207 | 29-Aug-04 | -21.74 | -145.52 | 970 | 13766 | M |
| PF04 | 208 | 29-Aug-04 | -21.74 | -145.52 | 1070 | 18299 | M |
| PF04 | 209 | 02-Sep-04 | -19.90 | -148.90 | 1100 | 19828 | M |
| PF04 | 210 | 22-Sep-04 | -12.09 | -144.74 | 950 | 12958 | M |
| PF04 | 211 | 25-Sep-04 | -12.21 | -144.56 | 920 | 11806 | F |
| PF04 | 212 | 26-Sep-04 | -12.22 | -144.53 | 1010 | 15478 | M |
| PF04 | 213 | 26-Sep-04 | -12.22 | -144.53 | 900 | 11077 | F |
| PF04 | 214 | 27-Sep-04 | -12.20 | -144.59 | 970 | 13766 | M |
| PF04 | 215 | 21-Oct-04 | -14.57 | -144.58 | 960 | 13358 | M |
| PF04 | 216 | 22-Oct-04 | -15.07 | -145.21 | 1020 | 15927 | M |
| PF04 | 217 | 23-Oct-04 | -15.11 | -146.09 | 970 | 13766 | M |
| PF04 | 218 | 26-Oct-04 | -15.11 | -146.09 | 1000 | 15037 | M |
| PF04 | 219 | 26-Oct-04 | -15.11 | -146.09 | 1060 | 17807 | M |
| PF04 | 220 | 27-Oct-04 | -15.11 | -146.09 | 980 | 14181 | M |
| NZ10 | 222 | 27-Apr-10 | -39.56 | 178.44 | 850 | 9384 | U |
| NZ10 | 223 | 27-Apr-10 | -39.56 | 178.44 | 720 | 5797 | U |
| NZ10 | 224 | 27-Apr-10 | -39.56 | 178.44 | 920 | 11806 | U |
| NZ10 | 225 | 27-Apr-10 | -39.56 | 178.44 | 730 | 6034 | U |
| NZ10 | 226 | 27-Apr-10 | -39.56 | 178.44 | 780 | 7313 | U |
| NZ10 | 227 | 27-Apr-10 | -39.56 | 178.44 | 820 | 8455 | U |
| NZ10 | 228 | 27-Apr-10 | -39.56 | 178.44 | 690 | 5124 | U |
| NZ10 | 229 | 27-Apr-10 | -39.56 | 178.44 | 770 | 7044 | U |
| NZ10 | 230 | 28-Apr-10 | -39.96 | 177.40 | 980 | 14181 | U |
| NZ10 | 231 | 28-Apr-10 | -39.96 | 177.40 | 780 | 7313 | U |
| NZ10 | 233 | 7-May-10 | -39.63 | 178.45 | 930 | 12182 | U |
| NZ10 | 234 | 7-May-10 | -39.63 | 178.45 | 940 | 12566 | U |
| NZ10 | 235 | 7-May-10 | -39.63 | 178.45 | 820 | 8455 | U |
| NZ10 | 236 | 7-May-10 | -39.63 | 178.45 | 850 | 9384 | U |
| NZ10 | 237 | 7-May-10 | -39.63 | 178.45 | 810 | 8159 | U |
| NZ10 | 238 | 7-May-10 | -39.63 | 178.45 | 790 | 7588 | U |
| NZ10 | 239 | 7-May-10 | -39.63 | 178.45 | 810 | 8159 | U |
| NZ10 | 240 | 7-May-10 | -39.63 | 178.45 | 780 | 7313 | U |
| NZ10 | 241 | 7-May-10 | -39.63 | 178.45 | 770 | 7044 | U |
| NZ10 | 242 | 7-May-10 | -39.63 | 178.45 | 740 | 6277 | U |
| NZ10 | 243 | 7-May-10 | -39.63 | 178.45 | 860 | 9708 | U |
| NZ10 | 244 | 7-May-10 | -39.63 | 178.45 | 800 | 7870 | U |
| NZ10 | 245 | 7-May-10 | -39.63 | 178.45 | 610 | 3584 | U |
| NZ10 | 246 | 7-May-10 | -39.63 | 178.45 | 800 | 7870 | U |
| NZ10 | 247 | 7-May-10 | -39.63 | 178.45 | 600 | 3416 | U |
| NZ10 | 249 | 9-May-10 | -39.73 | 178.38 | 780 | 7313 | F |
| NZ10 | 251 | 9-May-10 | -39.73 | 178.38 | 800 | 7870 | F |
| NZ10 | 252 | 9-May-10 | -39.73 | 178.38 | 1000 | 15037 | M |
| NZ10 | 253 | 9-May-10 | -39.73 | 178.38 | 730 | 6034 | U |
| NZ10 | 255 | 10-May-10 | -39.73 | 178.41 | 800 | 7870 | U |
| NZ10 | 256 | 10-May-10 | -39.73 | 178.41 | 800 | 7870 | F |
| NZ10 | 257 | 10-May-10 | -39.73 | 178.41 | 830 | 8758 | U |
| NZ10 | 258 | 10-May-10 | -39.73 | 178.41 | 810 | 8159 | U |
| NZ10 | 259 | 10-May-10 | -39.73 | 178.41 | 820 | 8455 | F |
| NZ10 | 260 | 10-May-10 | -39.73 | 178.41 | 820 | 8455 | U |
| NZ10 | 261 | 10-May-10 | -39.73 | 178.41 | 820 | 8455 | F |
| NZ10 | 262 | 10-May-10 | -39.73 | 178.41 | 960 | 13358 | F |
| NZ10 | 263 | 10-May-10 | -39.73 | 178.41 | 990 | 14605 | F |
| NZ10 | 264 | 15-May-10 | -40.29 | 177.22 | 770 | 7044 | U |
